# Books and Magazines

**Published:** 1907-09

**Authors:** 


### Books and Magazines.

THE PRINCIPLES AND PRACTICE OF DERMATOLOGY. For students and practitioners. By William Allen Pusey, A. M., M. D., Professor of Dermatology in the University of Illinois; Dermatologist to St. Luke's and Cook County Hospitals, Chicago, etc. One colored plate and three hundred and sixty-seven text illustrations. Cloth, $6. D. Appleton & Co., New York and London. 1907.

Dr. Pusey recognizes the fact that it is highly desirable in any study to be fully acquainted with the fundamental knowledge of the subject in order to obtain a satisfactory grasp of the special diseases, and has, therefore, given considerable space to "The Principles of Dermatology," as much as the importance of the subject demands, "Anatomy and Physiology of the Skin," "General Etiology," "Pathology," "Symptomatology" and "Treatment of Diseases of the Skin." "General Treatment" especially has been considered in full, because of its practical importance. The text of this work is particularly clear and lucid, and fully illustrated with 364 excellent illustrations. It would be impossible to mention all the good points in this book. It is enough to say that it is most exhaustive yet practical,—that in treatment is it particularly full,—and that the illustrations are, we believe, the best that have ever appeared in a book upon this subject.

---

Manual of Diseases of the Eye, by Charles H. May, Chief of Clinic and Instructor in Ophthalmology, College of Physicians and Surgeons, Medical Department, Columbia University, New York.—1890-1903; Ophthalmic Surgeon to the City Hospitals, Randall's Island, New York; Consulting Ophthalmologist to the French Hospital, to the Gouverneur Hospital, and to the Red Cross Hospital, New York; Adjunct Ophthalmic Surgeon to Mt. Sinai Hospital, New York, etc. Fifth edition, revised, with 362 original illustrations, with 22 plates, with 63 colored figures. Messrs. Wm. Wood & Co. 1907. The price of the book continues the same, $2 net.

In the following pages the author has endeavored to present a concise, practical, and systematic manual of the Diseases of the Eye, intended for the student and the general practitioner of medicine. The great difficulty in preparing a book of this sort is to say enough, but not too much. With this idea in view, the author has made the volume sufficiently comprehensive, up to date, and yet of limited size.

This restriction in size has been accomplished by omitting excessive detail, extensive discussion, and lengthy accounts of theories and rare conditions. The author has endeavored to give the fundamental facts of ophthalmology and to cover all that is essential in this branch of medicine, always keeping in mind that the book has been written for students and general practitioners. Space, therefore, has been allotted as the necessities of such readers require, estimated by an extended experience in teaching. Thus rare conditions have merely been mentioned; uncommon affections, of interest chiefly to the specialist, have been dismissed with a few lines; and common diseases, which the general practitioner is most frequently called upon to treat, have been described with comparative fulness.

The book is not recommended as a substitute for the larger works, but as a means of supplying a foundation to which further knowledge may be added by reference to more extensive and comprehensive text-books.

The illustrations, excepting those showing instruments, are original, and have been inserted wherever it seemed that they would be of value in elucidating the text. Those in colors represent the most common changes in the fundus, a knowledge of which is desirable in the treatment of general and nervous diseases as well as in ocular diagnosis, and thus practically supply an ophthalmoscopic atlas.

The demand for this work has been such as to necessitate since 1900 five American and several German, Dutch, French and Spanish editions, a fact that testifies to the great value and completeness of the publication.

---

THE PRACTICAL MEDICINE SERIES, comprising ten volumes on the year's progress in Medicine and Surgery, under the general editorial charge of Gustavus P. Head, M. D., Professor of Laryngology and Rhinology, Chicago Postgraduate Medical School.

I have received Volumes I, II and III of the Series of 1907. Volume I, General Medicine, by Billings and Salisbury; volume II, General Surgery, by J. B. Murphy; volume III, Eye, Ear, Nose and Throat, by Wood, Andrews and Head. Price of the ten volumes, $10. Those interested in special subjects can buy any volume separately they want; price, $1.25. Year-Book Publishing Co., Chicago. This well-known series contains a resume of all that is latest in the several departments of medicine culled from the best medical publications. They are "up to date," and in advance of more pretentious and larger works.

